# Potential Role of MicroRNA-375 as Biomarker in Human Cancers Detection: A Meta-Analysis

**DOI:** 10.1155/2017/1875843

**Published:** 2017-11-13

**Authors:** Jin Yan, Qiang She, Xiaoran Shen, Yifeng Zhang, Bingtuan Liu, Guoxin Zhang

**Affiliations:** ^1^Department of Gastroenterology, The First Affiliated Hospital with Nanjing Medical University, Nanjing, Jiangsu, China; ^2^The First Clinical Medical College, Nanjing Medical University, Nanjing, Jiangsu, China; ^3^Department of Gastroenterology, Yangzhou No. 1 People's Hospital, Yangzhou, Jiangsu, China; ^4^Department of Gastroenterology, Jiangyin People's Hospital, Jiangyin, Jiangsu, China

## Abstract

The association between circulating microRNA-375 (miR-375) expression and cancers has been studied; however, the results are inconsistent. We searched PubMed, Embase, and Web of Science for studies concerning the diagnostic value of miR-375 for cancer. The bivariate meta-analysis model was employed to summarize sensitivity, specificity, and diagnostic odds ratio (DOR) for miR-375 in the diagnosis of cancer. Summary receiver operating characteristic (SROC) curve analysis and the area under the curve (AUC) were also used to check the overall test performance. A total of 645 cancer patients and 421 cancer-free individuals from 12 studies were contained in this meta-analysis. The summary estimates revealed that the pooled sensitivity was 78% (95% confidence interval (CI): 64%–87%), the specificity was 74% (95% CI: 62%–84%), the DOR was 10.04 (95% CI: 6.01–16.77), and the AUC was 0.82 (95% CI: 0.79–0.85). In addition, we found that the diagnostic effect of miR-375 varies according to the race and cancer type. Our data suggest that miR-375 profiling has a potential to be used as a screening test for cancers but the specific race and cancer should be considered. More studies on the diagnostic value of miR-375 for cancer are needed in the future.

## 1. Introduction

Cancer has been a major public health problem and is considered as leading cause of death worldwide [1, 2]. As we all know, the prognosis for cancer patients is different depending on the time of diagnosis, but early cancer detection is still a compelling challenge for clinicians. Nowadays, the gold diagnosis standard for cancer is histological evaluation of biopsy. However, its usage is restricted in clinic for the invasive procedure and it fails to diagnose cancer at early stage [3]. Hence, there is an urgent need for noninvasive early detection biomarkers for cancer patients. In the present, several serum cancer biomarkers have been used in clinic, such as carcinoembryonic antigen (CEA), *α*-fetoprotein (AFP), and carbohydrate antigens 125 and 19-9 (CA 125 and CA 19-9) [4–7]. Nevertheless, these markers have inadequate sensitivity and specificity, limiting their application in the early cancer diagnosis. Thus, finding an effective noninvasive biomarker for early detection of cancer is in urgent need.

MicroRNAs (miRNAs), single-stranded RNAs with 19–25 nucleotides, play an important role in many physiological processes. They bind to specific mRNA targets via base pairing at the 3′-UTR, resulting in translational repression or degradation of these mRNAs [8]. Compared with noncancerous controls, miRNAs are differently expressed in tissues, serum, and plasm of cancer patients [9–12]. Blanco-Calvo et al. found that circulating miRNAs remain stable after incubation for up to 24 h at room temperature or after up to eight cycles of freezing and thawing and their stability makes miRNAs suitable for being cancer biomarkers [13, 14]. Among those cancer associated miRNAs, miR-375 was first identified as a pancreatic islet specific miRNA which regulated insulin secretion [15]. Further studies reported that miR-375 was involved in multiple types of cancer by targeting several important genes like AEG-1, YAP1, IGF1R, and PDK1 [16–19]. Additionally, plenty of studies have focused on the potential use of miR-375 as diagnosis biomarker. Komatsu et al. found that the circulating miR-21/miR-375 ratio might be a diagnostic marker in esophageal squamous cell carcinoma [20]. Moreover, miR-375, miR-25, and let-7f were found able to separate HBV-positive HCCs from healthy controls [21]. However, the expression tendency of miR-375 in different kinds of cancer was inconsistent and race was also involved in the reasons.

Choosing an appropriate biomarker for cancer detection should consider both diagnostic accuracy and prognostic relationship. In addition, we also need to take kind of disease and race into consideration. Nowadays, a meta-analysis suggests that miR-375 expression is associated with overall survival of cancer patients and could be a useful clinical prognostic biomarker [22]. However, the diagnostic accuracy of miR-375 for cancers was inconsistent in literature and the results of every paper were restricted because of limited enrolled patient number. Therefore, the aim of our study was to conduct a first meta-analysis to determine whether detection of miR-375 expression can be an effective biomarker for cancers and when it is most applicable.

## 2. Materials and Methods

### 2.1. Data Sources and Search Strategy

The meta-analysis followed the guidelines of the Preferred Reporting Items for Systematic Reviews and Meta-Analysis (PRISMA) statement and methods [23]. The following electronic databases PubMed, Embase, and Web of Science were searched. The key words used in the research were “serum or plasma or blood or circulating” and “microRNA-375 or miRNA-375 or miR-375” and “ROC curve or diagnosis or sensitivity or specificity.” Eligible studies published up to April, 2017 were screened. In addition, we manually searched the reference lists of eligible studies identified from the databases as well.

### 2.2. Inclusion and Exclusion Criteria

Two reviewers (J. Y and Q. S) checked the abstract of the studies and read the full-text if necessary to identify the final included studies. Disagreement was resolved by fully discussion and consulting with the third reviewer (X. R. S). Moreover, we turned to the original authors for data if necessary. The articles were considered eligible if they met the following criteria: (1) cancer diagnosis was based on histopathological confirmation and healthy people or patients with benign disease were served as the control group; (2) they detected miR-375 expression in serum, plasma, urine, and other body fluids; (3) the studies utilized a case-control design and contained sufficient published data to construct two-by-two tables and calculate the diagnostic accuracy; (4) they were published in English. In addition, articles were excluded if they were (1) review articles, meta-analysis, letters, commentaries, and abstracts presented in conferences; (2) duplicates or continued work of previous publications; (3) studies without complete data; (4) not in English.

### 2.3. Data Extraction

Data were retrieved from each study independently by two reviewers (J. Y and Q. S) including the following characteristics: the first author, year of publication, country and ethnicity of origin, sample type, cancer type, miR-375 change tendency, number of patients, diagnostic parameters, and other substantial information. Disagreements were solved by fully discussing with the third investigator (X. R. S).

### 2.4. Quality Assessment

To ensure the quality of the meta-analysis, all selected articles were scored and categorized according to the Quality Assessment of Diagnostic Accuracy Studies (QUADAS) [24]. The 14 items in QUADAS were assessed in all the included studies. Every item was assessed with “yes,” “no,” or “unclear.” An answer of “yes” will get one score, while the “no” or “unclear” will gain a score of zero. Two authors (J. Y and Q. S) reached a consensus on study quality assessment, and any disagreements were resolved through a discussion with the third author (X. R. S).

### 2.5. Statistical Analysis

All accuracy data from each study (true positives, false positives, true negatives, and false negatives) were extracted to obtain pooled sensitivity, specificity, positive likelihood ratio (PLR), negative likelihood ratio (NLR), diagnostic score, DOR, and their 95% CI. The value of a DOR ranges from 0 to infinity, with higher values indicating better discriminatory test performance [25]. The SROC curve and AUC were also gathered to evaluate the diagnosis accuracy of miR-375 in cancer. An AUC value close to 1.0 implies that the test has perfect discrimination, and an AUC value close to 0.5 suggests poor discrimination.

Moreover, threshold effect can cause heterogeneity between studies and was quantified by Spearman correlation analysis. The nonthreshold effect was assessed by the Cochran-*Q* method and the test of inconsistency index (*I*^2^). A low *p* value of less than 0.05 and high *I*^2^ value of more than 50% suggest presence of heterogeneity by nonthreshold effect. If the nonthreshold effect existed, meta-regression would be used to find out the sources. Finally, evidence of publication bias was analyzed by Deeks' funnel plot (*p* value less than 0.05 was considered a significant publication bias) [26]. Statistical analysis was conducted utilizing Stata 12.0 (Stata Corporation, College Station, TX, USA) and Meta-disc 1.4 (XI Cochrane Colloquium, Barcelona, Spain) software.

## 3. Results

### 3.1. Data Selection

**Figure 1:**
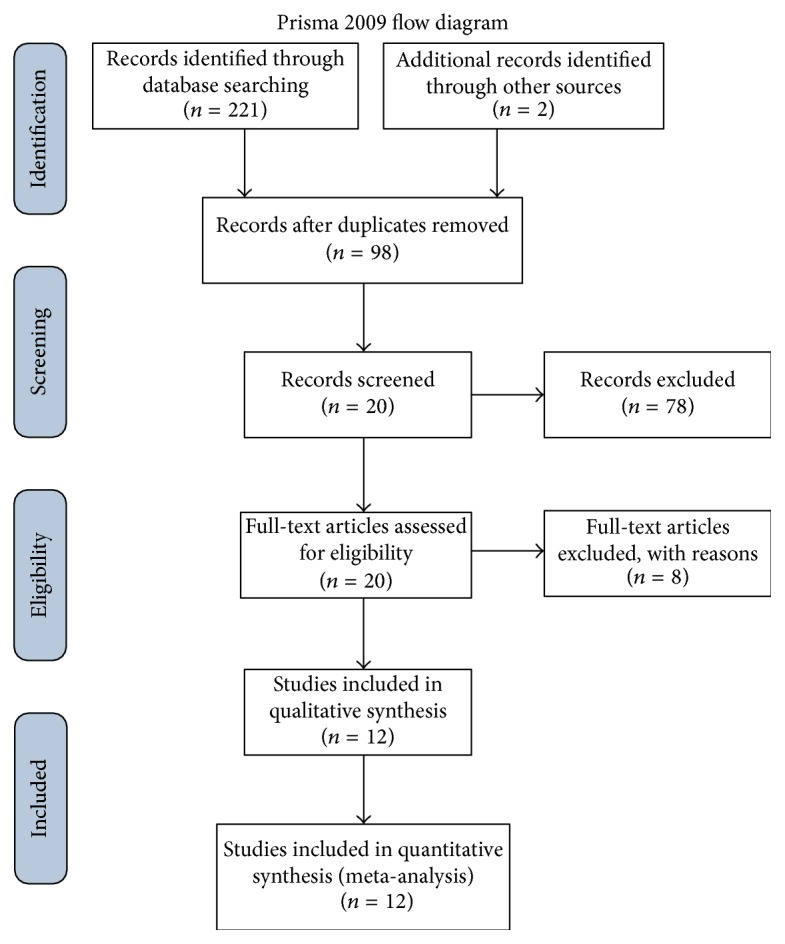
Flow chart of study selection based on the inclusion and exclusion criteria.

The initial search returned a total of 223 studies and 98 studies were left after duplicates were removed. We then screened titles and abstracts and excluded 7 reviews and 1 letter. Moreover, 2 studies were not in English, 3 were not human associated, 8 were not related to cancer, 24 were not about diagnosis effect, and 33 were not detected in body fluids. Of these remained 20 literatures; their full-text versions were retrieved and 8 of them were excluded due to lack of sufficient data and rational study design. Thus, 12 high-quality studies from independent research group met the eligibility criteria for this meta-analysis [20, 27–36] (Figure 1).

### 3.2. Study Characteristics and Quality Assessment

All of these eligible literatures were published from 2011 to 2017 accumulating 645 cancer patients and 421 controls. The cancer patients had been histopathologically confirmed, which is gold standard for cancer diagnosis. The control individuals were from volunteers who had never been diagnosed with a malignant tumor and patients with benign diseases. The study characteristics, including the first author, publication year, country, ethnicity, sample type, cancer type, miR-375 change tendency, number of patients, and diagnostic parameters, were listed in Table 1. In addition, the 12 studies were scored by QUADAS by two independent reviewers. Nine of the 11 studies had QUADAS scores more than 10, indicating the reliable foundation of our analysis (Tables 1 and 2).

### 3.3. Data Analysis

**Figure 2:**
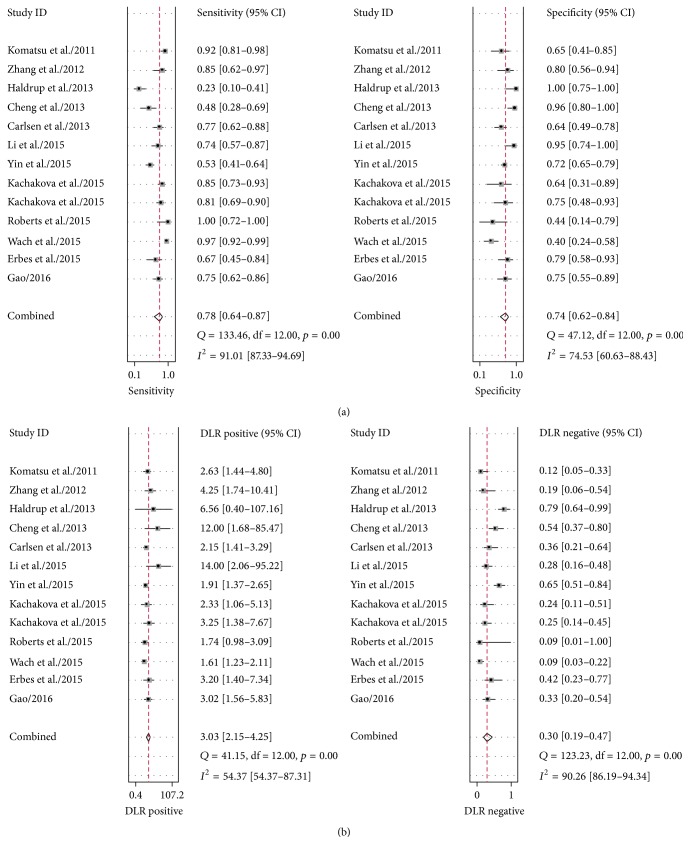
Forest plots of sensitivities, specificities, PLRs, and NLRs from test accuracy studies of miR-375 in the diagnosis of cancer.

**Figure 3:**
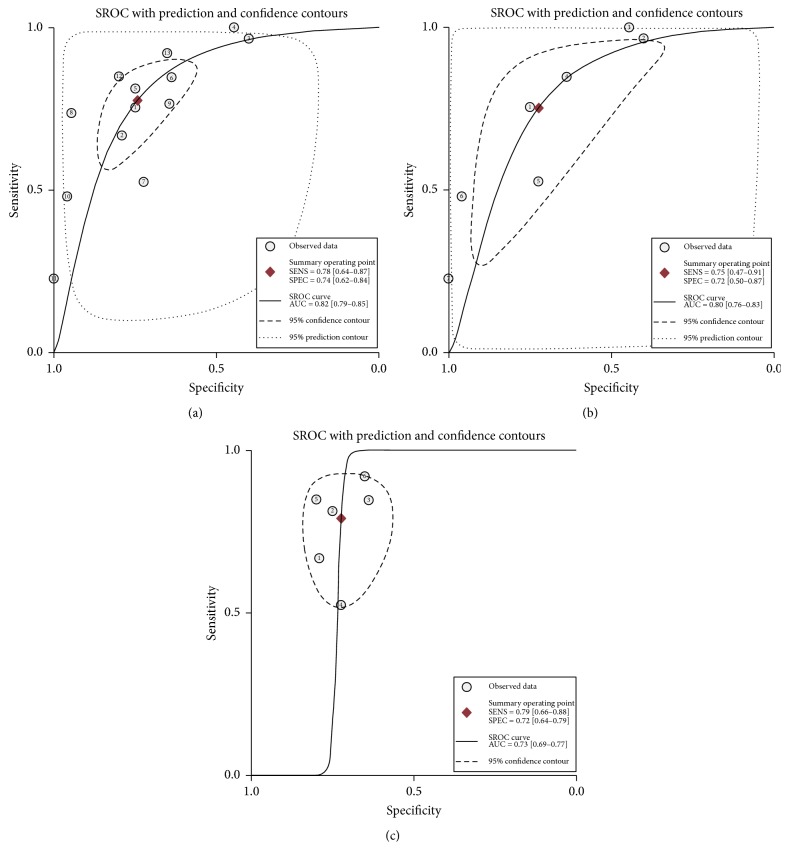
Summary receiver operating characteristic curve in all studies, miR-375 upregulated studies, and miR-375 downregulated studies.

**Figure 4:**
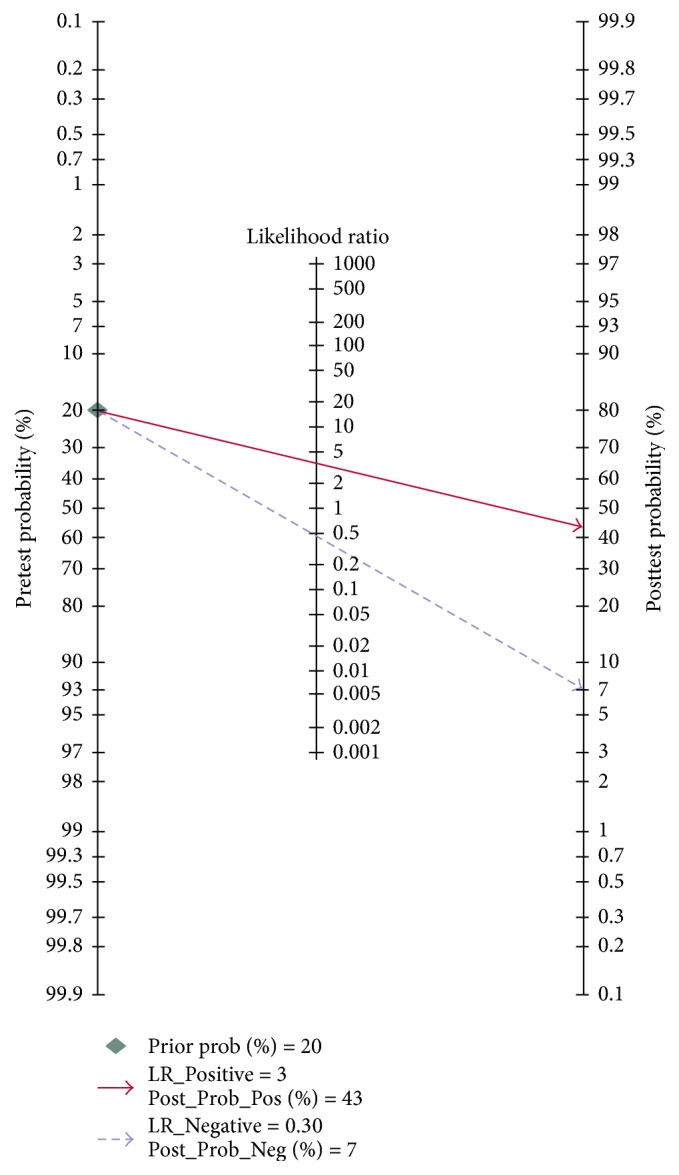
Fagan's nomogram in assessment of the test probabilities after miR-375 assay.

**Figure 5:**
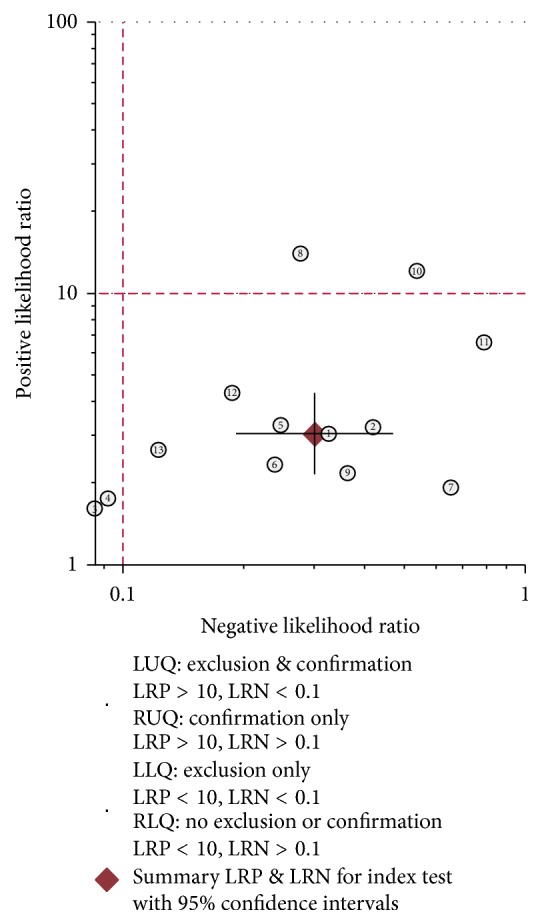
Pooled LNR and LPR of the diagnostic studies of circulating miR-375.

Heterogeneity in sensitivity and specificity were observed among these studies (*I*^2^ = 91.01% and *I*^2^ = 74.53%), which indicated significant heterogeneity (Figure 2). Therefore, the random effects model was selected in this study. For miR-375, the sensitivity, specificity, PLR, NLR, diagnostic score, and DOR of 13 studies in 12 included papers were performed by forest plots. A pooled sensitivity and specificity of miR-375 were 78% (95% CI: 64%–87%) and 74% (95% CI: 62%–84%) in the diagnosis of cancer patients, respectively (Figure 2(a)). Its PLR and NLR in diagnosis cancer were 3.0 (95% CI: 2.2–4.3) and 0.30 (95% CI: 0.17–0.47) separately (Figure 2(b)). The diagnostic score was 2.31 (95% CI: 1.79–2.82), and the DOR is 10.04 (95% CI: 6.01–16.77) (Figure S1 in Supplementary Material available online at https://doi.org/10.1155/2017/1875843). The SROC curve for the included studies was shown in Figure 3. The AUC was 0.82 (95% CI: 0.79–0.85), indicating a good diagnostic accuracy of miR-375 for cancer diagnosis. Furthermore, the posttest probability was calculated, and miR-375 harbored a pretest probability of 20% to have cancer. A positive result would improve posttest probability of having cancer to 43%, while a negative result would drop the posttest probability to 7% (Figure 4). Moreover, PLR > 10 or NLR < 0.1 indicated high accuracy. We showed summary point of PLR combined with NLR in the likelihood matrix (Figure 5) and found four studies were of high accuracy. The pooled diagnosis indexes of these four studies were sensitivity of 87% (95% CI: 59%–97%), specificity of 78% (95% CI: 38%–96%), PLR of 4.0 (95% CI: 1.2–13.8), NLR of 0.16 (95% CI: 0.06–0.46), DOR of 24 (95% CI: 10–61), and AUC of 0.90 (95% CI: 0.87–0.93). The sensitivity was increased and the NLR was decreased, but the results were influenced by limited study number.

The difference of miR-375 expression in cancer patients was inconsistent. Seven studies reported that miR-375 was upregulated in cancer patients and pooled sensitivity and specificity were 75% (95% CI: 47%–91%) and 72% (50%–87%), respectively. However, miR-375 was decreased in cancer patients compared to controls in the other six studies and pooled sensitivity and specificity were 79% (95% CI: 66%–88%) and 72% (64%–79%). In addition, the AUC was 0.80 (95% CI: 0.76–0.83) for upregulated six studies and 0.73 (95% CI: 0.69–0.77) for the downregulated group (Figures 3(b) and 3(c)). For prostate cancer the miR-375 was usually upregulated, but in Asian country the miR-375 was usually downregulated in gastroenterology cancers.

### 3.4. Threshold Effect and Heterogeneity

It is known that the heterogeneity between the studies can influence diagnosis accuracy. The threshold effect, one source of heterogeneity, is partly due to differences of sensitivity and specificity. Spearman correlation coefficient of sensitivity and specificity is a good way to evaluate the threshold effect [37]. In this meta-analysis, the Spearman correlation coefficient of sensitivity and 1 − specificity was 0.765 with a *p* value of 0.002 (*p* < 0.05), suggesting that there is heterogeneity from threshold effect. In addition, the nonthreshold effect is usually assessed by *I*^2^. Our results showed that *I*^2^ was more than 50%, suggesting that the heterogeneity by nonthreshold effect also existed among these studies. Then we performed the meta-regression based on the variables including publication year, country and ethnicity, sample type, cancer type, miR-375 change tendency, number of patients, and quality score, to explain this heterogeneity. Unfortunately, metaregression analysis indicated that above variables were not the sources of heterogeneity for this study.

### 3.5. Publication Bias

**Figure 6:**
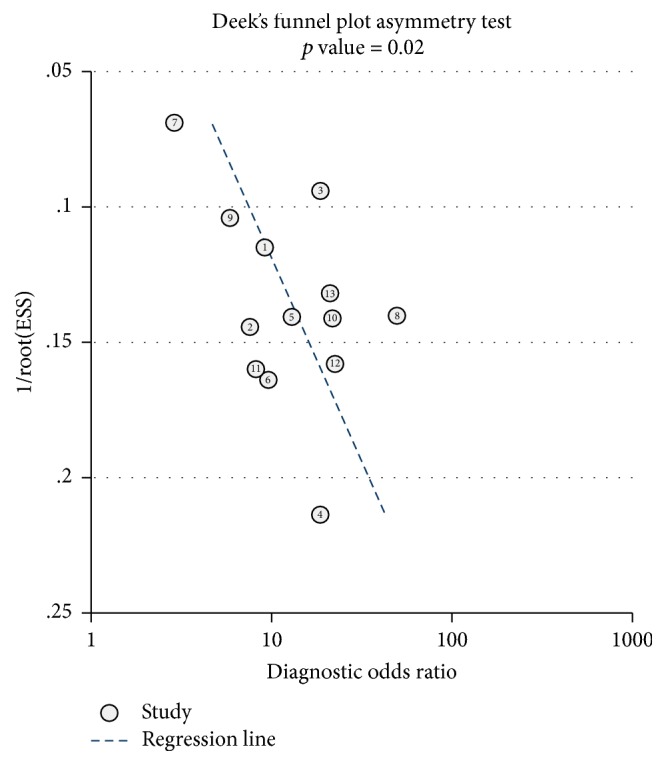
Deeks' funnel plots asymmetry test with regression line to explore publication bias.

To assess publication bias in this study, the included studies were evaluated using Deeks' test. As shown in Figure 6, Deeks' funnel plot was asymmetric, and the *p* value was 0.02. Thus, there was significant publication bias in this meta-analysis.

## 4. Discussion

As we know, this is the first report evaluating the diagnostic efficacy of circulating miR-375 in cancers. In the present study, we found that circulating miR-375 could discriminate cancer from controls and yielded an AUC of 0.82 (95% CI: 0.79–0.85) with a sensitivity of 78% (95% CI: 64%–87%) and a specificity of 74% (95% CI: 62%–84%). An AUC of 0.93 to 1 is considered to be of very good diagnosis effect and 0.75 to 0.92 is good [38]. This result of 0.82 suggests that miR-375 is a good potential noninvasive biomarker for cancer. The DOR is a single indicator of test accuracy that combines sensitivity and specificity. We identified that the pooled DOR was 10.04 (95% CI: 6.01–16.77), indicating that the overall accuracy of the miR-375 test for detecting cancer was relatively high. The PLR and NLR are more clinically meaningful for measures of diagnostic accuracy. The pooled PLR and NLR were 3.0 (95% CI: 2.2–4.3) and 0.30 (95% CI: 0.17–0.47), respectively. The PLR value of 3.0 suggests that patients with cancer have an approximately 3.0-fold higher chance of being miRNA-375 differently expressed compared to control patients without cancer. The NLR value of 0.30 means that the probability of the person having cancer is 30% if the miR-375 is abnormal. The miR-375 was reported to be increased in some studies. On the contrary, some literatures implied miR-375 was downregulated in plasm of cancer patients and the diagnosis accuracies were relatively similar. So more studies with larger number of cases and controls are indeed needed to assess the change tendency of miR-375.

Heterogeneity is a potential problem in interpreting the results of meta-analysis. Primary causes of heterogeneity in test accuracy studies are threshold effect, nonthreshold effect, and publication bias [39]. The Spearman correlation coefficient of sensitivity and 1 − specificity is 0.765 (*p* = 0.002), which indicates that there is obvious heterogeneity from threshold effects. Differences in sensitivities and specificities due to different cut-offs or thresholds used in different studies to define a positive or negative test result can lead to threshold effects. Although the detecting methods for miR-375 are all based on real-time quantification PCR (qPCR), there are no unified primers for qPCR analysis. Therefore, different laboratories take different measures to quantify the miR-375, which may contribute to sources of heterogeneity [38]. In our meta-analysis, the *p* value of Deeks' funnel plot was less than 0.05, so there is also a risk that publication bias might adversely affect the reliability of the result. These results indicate that more studies with consistent procedure standard are needed.

Proper and noninvasive tumor biomarkers are essential to early cancer detection and diagnosis. miRNAs have been reported to be involved in the development of tumor as a regulator in gene expression [40]. In addition, recent studies have confirmed that miRNAs were released from broken cells and entered into the circulation system including blood and other body fluid. Furthermore, endogenous plasma miRNAs were reported to exist in a form that is resistant to plasma RNase activity, implying miRNAs in plasma remain intact and are stable for detection [41]. At present, many miRNAs have been proved to be a biomarker for cancer detection. Zeng et al. conducted a meta-analysis and found miR-21 has potential diagnostic value with a moderate sensitivity and specificity for gastric cancer [38]. In addition, more and more attention has been focused on miR-375 and a meta-analysis conducted in 2014 has proven miR-375 was significantly related to cancer overall survival. When we seek novel cancer biomarker, prognostic relationship and diagnostic efficiency must be taken into account. They assess the cancer biomarker from two different aspects and with different evaluation indexes. However, the diagnostic efficiencies are inconsistent due to limited case number. To our knowledge, we are the first meta-analysis to evaluate if miR-375 could act as a novel biomarker to diagnosis cancer.

Although its results are promising, this meta-analysis still has limitations. Firstly, the study size obtained in this meta-analysis is relatively small. Therefore, further validations of miR-375 in large cohort and independent studies are needed. Secondly, although we have tried our best to cover all the involved literatures by a comprehensive method, we may still miss some of them during the screen process. Thirdly, only articles written in English were included in this meta-analysis, and articles written in other languages, unpublished data, and ongoing studies were not included, which may contribute to publication bias in our meta-analysis. Finally, a lack of access to the original data from the included studies limited our ability to perform meta-analysis on miR-375 diagnosis effect.

In conclusion, our meta-analysis suggests that miR-375 has potential diagnostic value with reasonable specificity and sensitivity for cancer. Larger scale prospective studies are needed in future to further validate its diagnostic effect. If validated in a large scale study, miR-375 might be useful as a noninvasive screening tool for clinical practice of cancer.

## Supplementary Material

Figure S1: Forest plots of diagnosis score and DOR from test accuracy studies of miR-375 in the diagnosis of cancer.

## Figures and Tables

**Table 1:** Characteristics of 11 articles included in our study that reported on using miR-375 as diagnostic biomarkers of various cancers.

Author/year	Country/ethnicity	Sample	Cancer type	change in cancer	Case/control	TP	FP	FN	TN	Quality score (QUADAS)
Gao/2016	China/Asian	Plasma	Prostate cancer (versus benign prostate hyperplasia)	Up	57/28	43	7	14	21	8
Erbes et al./2015	Germany/Caucasian	Urine	Breast cancer	Down	24/24	16	5	8	19	8
Wach et al./2015	Germany/Caucasian	Serum	Prostate cancer (versus benign prostate hyperplasia)	Up	146/35	141	21	5	14	10
Roberts et al./2015	Australia/Caucasian	Ejaculate	Prostate cancer	Up	11/9	11	5	0	4	10
Kachakova et al./2015	Bulgaria/Caucasian	Serum	Prostate cancer (versus benign prostate hyperplasia)	Down	59/16	48	4	11	12	11
			Prostate cancer	Down	59/11	50	4	9	7	11
Yin et al./2015	China/Asian	Serum	Hepatocellular carcinoma	Down	78/156	41	43	37	113	11
Li et al./2015	China/Asian	Plasma	Esophageal squamous cell carcinoma	Up	38/19	28	1	10	18	11
Carlsen et al./2013	Denmark/Caucasian	Plasma	Pancreatic ductal adenocarcinoma	Up	47/45	36	16	11	29	10
Cheng et al./2013	America/Caucasian	Serum	Metastatic prostate cancer	Up	25/25	12	1	13	24	7
Haldrup et al./2013	Japan/Asian	Serum	Prostate cancer (versus benign prostate hyperplasia)	Up	31/13	7	0	24	13	10
Zhang et al./2012	China/Asian	Serum	Distal gastric adenocarcinoma	Down	20/20	17	4	3	16	11
Komatsu et al./2011	Japan/Asian	Plasma	Esophageal squamous cell carcinoma	Down	50/20	46	7	4	13	10

TP: true positives, FP: false positives, TN: true negatives, and FN: false negatives.

**Table 2:** QUADAS assessment for the eligible studies.

Enrolled study	Items of QUADAS													
	(1)	(2)	(3)	(4)	(5)	(6)	(7)	(8)	(9)	(10)	(11)	(12)	(13)	(14)
Gao/2016	N	Y	Y	Y	Y	Y	Y	Y	N	U	U	Y	N	N
Erbes et al./2015	N	Y	Y	Y	U	U	Y	Y	N	U	Y	Y	U	Y
Wach et al./2015	N	Y	Y	Y	Y	Y	Y	Y	U	U	Y	Y	U	Y
Roberts et al./2015	N	Y	Y	Y	Y	Y	Y	Y	Y	U	Y	U	U	Y
Kachakova et al./2015	N	Y	Y	Y	Y	Y	Y	Y	Y	U	Y	Y	U	Y
Yin et al./2015	N	Y	Y	Y	Y	Y	Y	Y	Y	U	Y	Y	U	Y
Li et al./2015	N	Y	Y	Y	Y	Y	Y	Y	Y	U	Y	Y	U	Y
Carlsen et al./2013	N	Y	Y	Y	Y	Y	Y	Y	U	U	Y	Y	U	Y
Cheng et al./2013	N	Y	Y	Y	N	U	Y	Y	N	U	Y	U	U	Y
Haldrup et al./2013	N	Y	Y	Y	Y	Y	Y	Y	Y	U	Y	U	U	Y
Zhang et al./2012	N	Y	Y	Y	Y	Y	Y	Y	Y	U	Y	Y	U	Y
Komatsu et al./2011	N	Y	Y	Y	Y	Y	Y	Y	Y	U	Y	U	U	Y

(1) Was the spectrum of patients representative of the patients who will receive the test in practice? (2) Were selection criteria clearly described? (3) Is the reference standard likely to correctly classify the target condition? (4) Is the time period between reference standard and index test short enough to be reasonably sure that the target condition did not change between the two tests? (5) Did the whole sample or a random selection of the sample receive verification using a reference standard of diagnosis? (6) Did patients receive the same reference standard regardless of the index test result? (7) Was the reference standard independent of the index test (i.e., the index test did not form part of the reference standard)? (8) Was the execution of the index test described in sufficient detail to permit replication of the test? (9) Was the execution of the reference standard described in sufficient detail to permit its replication? (10) Were the index test results interpreted without knowledge of the results of the reference standard? (11) Were the reference standard results interpreted without knowledge of the results of the index test? (12) Were the same clinical data available when test results were interpreted as would be available when the test is used in practice? (13) Were uninterpretable/intermediate test results reported? (14) Were withdrawals from the study explained?
