# Supplementary material for: Potential Role of MicroRNA-375 as Biomarker in Human Cancers Detection: A Meta-Analysis
Source: Biomed Res Int. 2017 Nov 13;2017:1875843. doi: 10.1155/2017/1875843 (PMC5702930; doi:10.1155/2017/1875843)

Supporting Information

Figure S1. Forest plots of diagnosis score and DOR from test accuracy studies of miR-375 in the diagnosis of cancer.

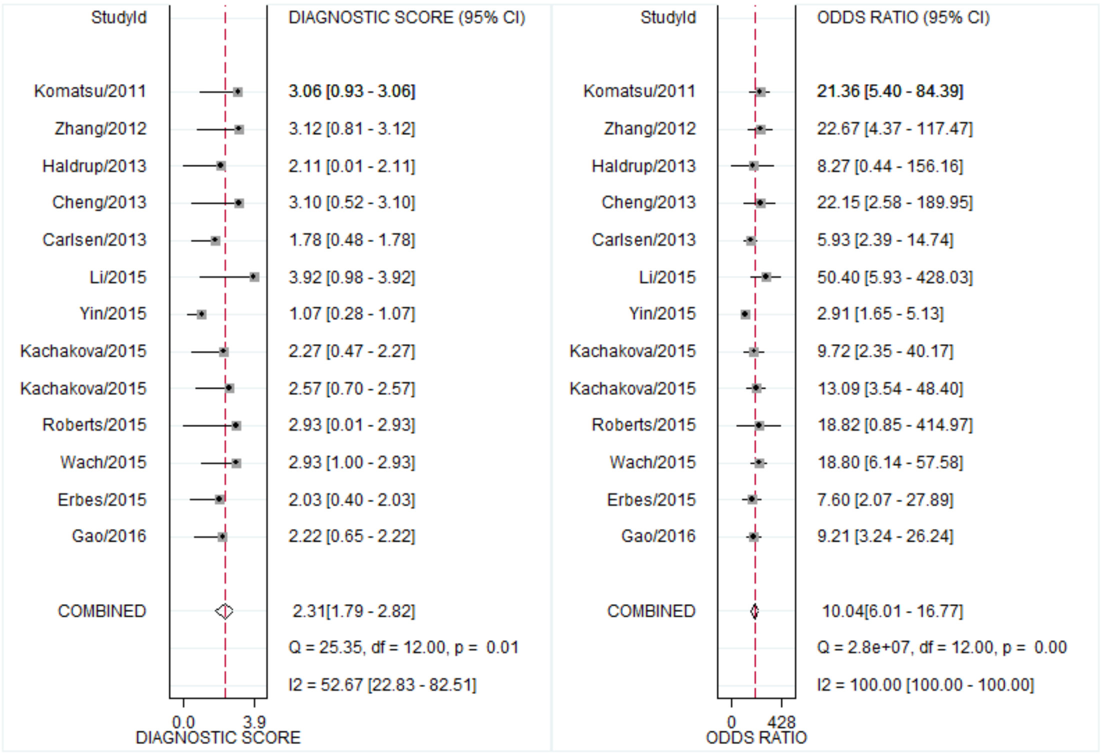

Supplement: Supplementary file 1 — Figure S1: Forest plots of diagnosis score and DOR from test accuracy studies of miR-375 in the diagnosis of cancer. [file 1875843.f1.pdf]
